# Differential expression of galectin-3, CK19, HBME1, and Ret oncoprotein in the diagnosis of thyroid neoplasms by fine needle aspiration biopsy

**DOI:** 10.4103/1742-6413.55894

**Published:** 2009-09-18

**Authors:** Husain A. Saleh, Jining Feng, Farah Tabassum, Opada Al-Zohaili, Muji Husain, Tamara Giorgadze

**Affiliations:** Department of Pathology, Wayne State University, Sinai-Grace Hospital, Detroit, MI, USA; 1 Department of Pathology, Harper University Hospital, Detroit Medical Center, Detroit, MI, USA; 2 Department of Medicine, Endocrinology Division, Wayne State University, Sinai-Grace Hospital, Detroit Medical Center, Detroit, MI, USA

**Keywords:** CK19, fine needle aspiration, galectin-3, HBME-1, immunohistochemical, Ret oncoprotein, thyroid

## Abstract

**Background:**

Fine needle aspiration biopsy (FNAB) is a common and excellent procedure for the evaluation of thyroid lesions that require surgical resection. At times, the FNAB diagnosis can be difficult, particularly of follicular-patterned lesions. Previous studies have shown that some immunohistochemical (IHC) markers may be helpful in establishing more accurate diagnosis. In this study, our goal was to evaluate four of the recently investigated markers in differentiating benign from malignant thyroid nodules on FNABs.

**Materials and Methods:**

We performed IHC staining of galectin-3, Ret oncoprotein (Ret), HBME-1, and cytokeratin 19 (CK19), on cell block sections of thyroid FNAB cases that had corresponding surgical resections. They included 44 benign lesions (37 hyperplastic or cellular nodules, HN; and 7 follicular adenomas, FA) and 27 malignant tumors (6 follicular carcinoma, FC; 19 classic papillary carcinoma, PTC; and 2 follicular variant of papillary carcinoma, FVPC). The stains were done according to the standard avidin–biotin–peroxidase method.

**Results:**

Statistical analysis showed that immunoexpression was significantly higher in the malignant group for all four markers. The sensitivity for positive expression for all benign lesions versus malignant tumors was as follows: 10/44 (22.7%) versus 25/27 (92.6%) for galectin-3; 14/44 (31.8%) versus 23/27 (85%) for Ret; 12/44 (27.3%) versus 24/27 (88.8%) for HBME-1; and 13/44 (29.5%) versus 23/27 (85%) for CK19. The sensitivity and specificity was highest for galectin-3 (92.6% and 77.3%, respectively) followed by HMBE-1 (88.9% and 72.7%, respectively). When combining the markers' expressions, the panel of galectin-3 + HBME-1 showed the highest sensitivity and specificity (90.7% and 75%, respectively), but this was, however, lower than galectin-3 alone (92.3% and 77.3%, respectively).

**Conclusion:**

We conclude that galectin-3 is the best single marker in differentiating benign from malignant thyroid lesions with the highest sensitivity and specificity. The galectin-3 + HBME-1 was the best combination for distinguishing benign from malignant lesions. Because they were the best two independent and combined markers, we recommend the use of the galectin-3 + HBME-1 panel to enhance the diagnostic accuracy of follicular-patterned thyroid lesions on FNABs.

## INTRODUCTION

Fine needle aspiration biopsy (FNAB) is a well-established procedure in the preoperative evaluation of thyroid nodules.[1] It is simple, cost effective, and practical with negligible side effects. Over the years, the use of FNA for the evaluation of thyroid nodules has resulted in a 50% decrease in surgical intervention with a 3-fold increase in finding cancer in the surgically resected specimens.[1,2] Nonetheless, there are well-known limitations in the role of thyroid FNA, most importantly its inability to differentiate benign from malignant follicular neoplasms, since this diagnosis rests on the histologic identification of capsular penetration and/or vascular invasion.[3–5] Another dilemma encountered on thyroid FNA specimens is the difficulty of distinguishing follicular variant of papillary carcinoma (FVPC) from follicular neoplasm.[6–9]

New techniques have been introduced to thyroid FNA procedure to enhance its diagnostic yield and improve the accuracy. Following a widespread success in the application of liquid-based monolayer preparation methodology in gynecologic (Pap test) and nongynecologic cytology specimens, this technique was introduced to FNA specimens of various organs including the thyroid.[10]. In fact, during the past several years, this technique (particularly ThinPrep method) has gained a growing acceptance and popularity by the clinical physicians and cytopathologists due to easy collection, preparation, and morphologic interpretation.[10,11] Most studies have demonstrated a decreased rate of unsatisfactory samples and improved diagnostic accuracy by using this method.

In spite of these improvements, there remain some limitations, inherent to thyroid FNA, including difficulties in cytodiagnosis of follicular-derived lesions. For this reason, attention has shifted to identifying molecular or immunohistochemical (IHC) markers that can help in separating adenomatous colloid nodules or follicular adenoma (FA) from follicular carcinoma (FC) on one hand, and FVPC from follicular neoplasm on the other hand.[12] During the past several years, a growing number of IHC markers have been tested on histologic and, to a lesser extent, on FNA samples with variable success rates.[13–26] In the majority of studies, the evaluation of IHC markers in thyroid FNA specimens was done on cell block sections;[14,27] however, some investigators showed that the staining can be successfully applied on unstained liquid-based slides (usually ThinPrep slides)[11,28,29] or destained Diff-Quik, hematoxylin and eosin, or Papanicolaou smears.

Galectin-3 is a component of the beta-galactoside-binding lectins. It is involved in cell-to-cell and cell-to-matrix modulation and appears to have a role in cell growth and differentiation. It is expressed in a high proportion of thyroid carcinomas, especially classic papillary carcinoma (PTC) and, therefore, it probably plays a significant role in the malignant transformation of thyroid follicular cells.[16,19,20,30] HBME-1 is a monoclonal antibody directed against an antigen of the microvillous surface of mesothelioma cells. It has been found positive in thyroid malignancies, especially PTC, and negative in benign lesions.[24,26,30] The Ret oncoprotein shows no reactivity in thyroid follicular cells, unless its rearranged form (reflecting the Ret gene activation) is present. Ret IHC expression has been found in thyroid carcinomas, particularly PTC. CK19 has also been shown to be helpful, and several studies demonstrated strong and diffuse positivity in malignant thyroid tumors including classic PTC, FVPC, and FC. However, other studies showed variable results and a few authors demonstrated that CK19 expression is mostly focal and weak in benign hyperplastic nodules (HN), FA, and FC.[17,18,30]

In this study, our goal was to evaluate the usefulness of using four of the most promising IHC markers (galectin-3, HBME-1, CK19, and Ret oncoprotein) on FNA cell block sections of thyroid lesions to identify the most helpful marker(s) in separating benign from malignant nodules. We also aimed at identifying the best panel of these four markers for providing the highest sensitivity and specificity for accurate diagnoses.

## MATERIALS AND METHODS

Forty-four benign and 27 malignant thyroid FNA cases confirmed by subsequent surgical resection specimens, during the period of 2000–2008, were collected from the archive of the pathology department at the Detroit Medical Center, Detroit, Michigan. The 44 benign FNA cases included 37 cases of hyperplastic colloid nodules or cellular colloid nodules (HN), and 7 cases of follicular or Hurthle cell adenomas (FA/HA). The 27 FNA cases of malignant tumors included 6 cases of FC, 19 cases of PTC, and 2 cases of FVPC. For simplicity and practical clinical considerations, we divided these lesions into two groups: benign (including nonneoplastic and neoplastic) and malignant.

The histologic diagnosis was based on the published WHO criteria. All the cell block samples were collected at the time of the FNA procedure by rinsing the aspiration needle in Saccomano's cytology fixative (QC Sciences, Glen Allen, VA, USA) or Cytolyt solution (Cytyc Corporation, Boxborough, MA, USA). A cell button was made by spinning the tube, discarding the supernatant, and then adding thromboplastin. The cell button was then fixed in 10% buffered zinc formalin, and embedded in paraffin to make a paraffin block. Four-micron-thick sections stained with hematoxylin and eosin (H and E) were prepared for the routine histological examination. Immunohistochemical staining was performed using four commercially available selected markers that have been shown in recent studies to be favorably expressed in malignant thyroid tumors. Table 1 shows the characteristics of the used antibodies including CK19, HBME-1, and galectin-3 and Ret oncoprotein. All immunohistochemical stains were performed on a Ventana Benchmark automated strainer. After standard protocols for deparaffinization of the 4-μm-thick sections, and microwave antigen retrieval, the tissue sections were incubated with the selected commercial monoclonal antibodies diluted at 1:100, except for Ret oncoprotein that used 1:40 dilution, for 32 min. The staining was completed using the avidin–biotin complex detection method. The positive controls included histiocytes for galectin-3, mesothelioma cells for HBME-1, skin for CK19, and a known case of PTC for Ret oncoprotein. The stained slides were examined by three pathologists (HS, TG, JF) blindly and independently without knowing the original cytologic or histologic diagnosis. A case was considered positive when 10% or more of the lesional cells showed cytoplasmic or nuclear staining. The staining results were then correlated with the original cytologic diagnoses and data tabulated.

**Table 1:** Characteristics of the antibodies used

*Antibody*	*Clone*	*Dilution*	*Antigen retrieval*	*Company*
CK19	RCK108	1:100	HIER EDTA buffer	Dako Corp.
HBME-1	HBME-1	1:50	HIER EDTA buffer	Dako Corp.
Galectin-3	9C4	1:100	HIER citrate buffer	Leica Microsystems
Retoncoprotein	3F8	1:40	HIER EDTAbuffer	Leica Microsystems

Dako Corporation, Carpentaria, CA, USA; Leica Microsystems, Bannockburn, IL, USACK19, cytokeratin 19

### Interpretation and analysis

Galectin-3 displayed cytoplasmic and nuclear staining; HBME1 staining was cytoplasmic with membranous and luminal accentuation; CK19 staining was diffusely cytoplasmic and Ret oncoprotein staining was cytoplasmic. A lesion was considered positive for a marker when 10% or more of the cells showed characteristic reactivity for the immunostain. Sensitivity, specificity, positive predictive value (PPV), and negative predictive value (NPV) were calculated for each of the markers and for different combinations of these markers in the benign versus malignant, benign nonneoplastic versus malignant and benign neoplastic versus malignant groups. The calculation was done using a known statistical computer software program (SPSS 10.0 for Windows; SPSS Inc., Chicago, IL, USA).

## RESULTS

**Figure 1:**
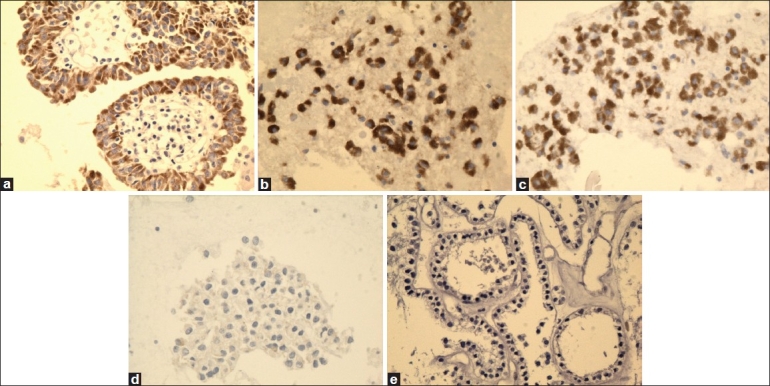
High magnification of galectin-3 immunostain showing reactivity in PTC (a), FVPC (b), and FC (c), and negative reaction for FA (d) and HN (e) (×40, galectin-3 immunostain)

**Figure 2:**
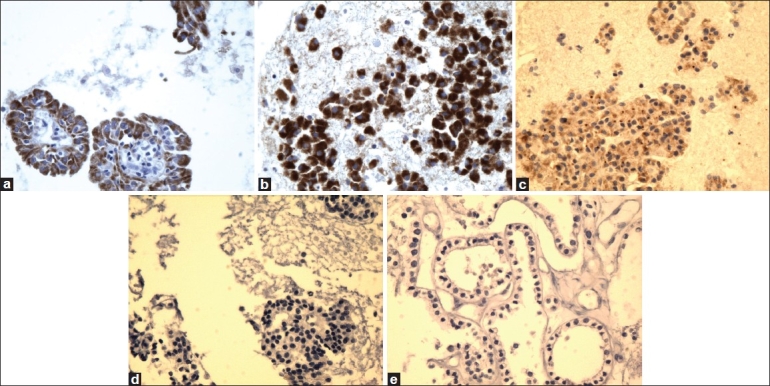
High magnification of Ret oncoprotein immunostain showing reactivity in PTC (a), FVPC (b), and FC (c), and negative reaction for FA (d) and HN (e) (×40, Ret oncoprotein immunostain)

**Figure 3:**
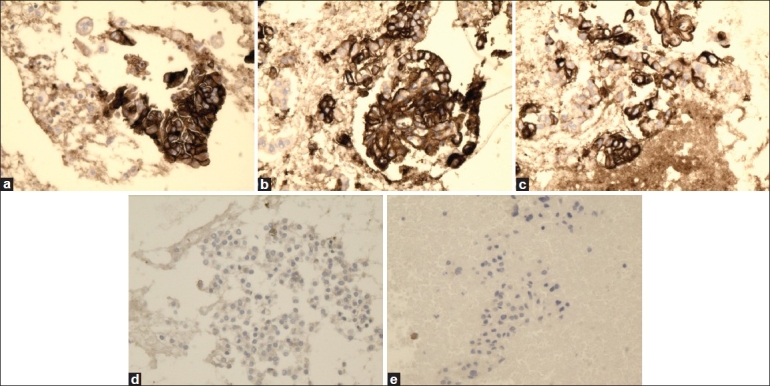
High magnification of HBME-1 immunostain showing reactivity in PTC (a), FVPC (b), and FC (c), and negative reaction for FA (d) and HN (e) (×40, HBME-1 immunostain)

**Figure 4:**
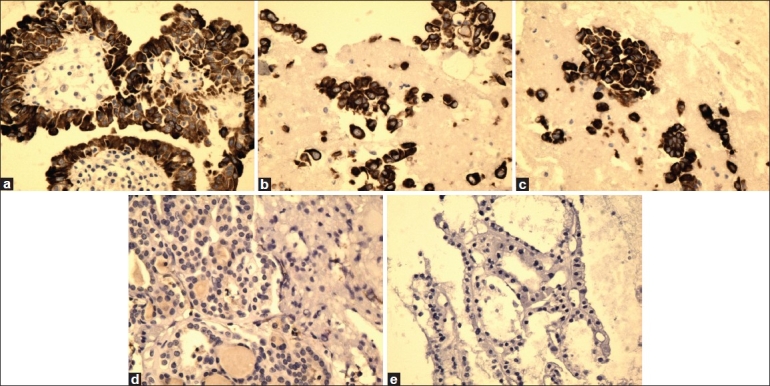
High magnification of CK19 immunostain showing reactivity in PTC (a), FVPC (b), and FC (c), and negative reaction for FA (d) and HN (e) (×40, CK19 immunostain)

Table 2 shows a summary of the cytologic diagnoses of the cases with the staining results for each group of the thyroid lesions. It was clear that malignant lesions have a significantly higher reactivity rate for all markers compared to the benign lesions. Only 10/44 (22.7%) benign lesions were positive for galectin-3 compared to 25/27 (92.6%) of the malignant lesions [Figure 1]. With Ret, 14/44 (31.8%) benign lesions were positive, while 23/27 (85%) malignant lesions were immunoreactive [Figure 2]. HBME-1 showed a clear difference in immunoreactivity between the two groups, with 12/44 (27.3%) positive benign lesions and 24/27 (88.8%) positive malignant tumors [Figure 3]. For CK19, 13/44 (29.5%) benign lesions were immunoreactive compared to 23/27 (85%) of the malignant lesion [Figure 4].

**Table 2:** Correlation of immunohistochemical staining results with the cytological diagnosis of thyroid lesions

*Markers*	*Benign (44)*			*Malignant neoplastic (27)*			
		
	*Benign nonneoplastic*	*Benign neoplastic*	*All benign (44 (%))*				
							
	*NH (37)*	*FA/HA (5/2 = 7)*		*FC (6)*	*PTC (19)*	*FVPC (2)*	*27 (%)*
Gal-3	8	2	10 (22.7)	5	18	2	25 (92.6)
RET	10	4	14 (31)	4	17	2	23 (85)
HBME-1	9	3	12 (27.3)	5	17	2	24 (88.8)
CK19	10	3	13 (29.5)	5	17	1	23 (85)

Gal-3-Galectin-3; HN-Hyperplastic or adenomatous colloid nodule; FA-Follicular adenoma; HA-Hurthle cell adenoma; FC-Follicular carcinoma; PTC-Papillary thyroid carcinoma; FVPC-Follicular variant of papillary carcinoma

Table 3 shows that galectin-3 has the best sensitivity and specificity for separating benign from malignant thyroid lesions with 92.6% and 77.3% values, respectively. HBME-1 had the second best sensitivity and specificity with values of 88.9% and 72.7%, respectively. The sensitivity and specificity was 85.2% and 68.2%, respectively, for Ret; and 85.2% and 70.5%, respectively, for CK19. Table 3 also depicts the PPV and NPV for all markers. The highest values were seen with galectin-3 at 71.4% and 94.4% for PPV and NPV, respectively. Again, HBME-1 demonstrated second best values for PPV and NPV at 66.7% and 91.4%, respectively. These results were, however, not statistically significant with *P* value > 0.05 for all four markers.

**Table 3:** Comparison of sensitivity, specificity, PPV, and NPV of individual marker expression in all benign versus malignant thyroid lesions

*Markers*		*Negative*	*Positive*	*Total*	*Combo values*	
Gal-3	Benign	34	10	44	Sensitivity	0.926
	Malignant	2	25	27	Specificity	0.773
					PPV	0.714
Total		36	35	71	NPV	0.944
Ret	Benign	30	14	44	Sensitivity	0.852
	Malignant	4	23	27	Specificity	0.682
					PPV	0.622
Total		34	37	71	NPV	0.882
HBME-1	Benign	32	12	44	Sensitivity	0.889
	Malignant	3	24	27	Specificity	0.727
					PPV	0.667
Total		35	36	71	NPV	0.914
CK19	Benign	31	13	44	Sensitivity	0.852
	Malignant	4	23	27	Specificity	0.705
					PPV	0.639
Total		35	36	71	NPV	0.886

Gal-3-Galectin-3; Ret-Ret oncoprotein; CK 19-Cytokeratin 19; PPV-Positive predictive value; NPV-Negative predictive value

Tables 2 and 4 compare markers immunoexpressions between the two groups of benign and malignant tumors. Clearly, all markers had a higher rate of immunoexpression in malignant tumors (PTC, FVPC, and FC) compared to benign tumors (FA/HA). The markers were expressed in approximately half the cases of benign tumors compared to over 85% expression in the malignant tumors (92.6% for galectin-3). The sensitivity and specificity of the markers for the diagnosis of benign and malignant tumors were again highest with galectin-3 at 92.6% and 71.4%, respectively.

Tables 2 and 5 also demonstrate a clear difference in immunoexpression of all markers between nonneoplastic and malignant lesions. Only about a quarter of the nonneoplastic cases were positive for these four markers compared to more than 85% in the malignant lesions. However, the sensitivity and specificity for immunoexpression of these markers were not as good as for benign neoplastic versus malignant [Table 5].

**Table 4:** Sensitivity, specificity, PPV, and NPV of benign neoplastic (adenomas) versus malignant thyroid tumors for each of the IHC markers

*Markers*	*Sensitivity*	*Specificity*	*PPV*	*NPV*
Gal-3	0.926	0.714	0.926	0.714
Ret	0.852	0.571	0.885	0.500
HBME-1	0.889	0.429	0.857	0.500
CK19	0.852	0.429	0.852	0.429

Gal-3-Galectin-3; Ret-Ret oncoprotein; CK 19-Cytokeratin 19; PPV-Positive predictive value; NPV-Negative predictive value

**Table 5:** Sensitivity, specificity, PPV, and NPV of benign nonneoplastic versus malignant thyroid lesions for each of the IHC markers

*Markers*	*Sensitivity*	*Specificity*	*PPV*	*NPV*
Gal-3	0.794	0.784	0.771	0.806
RET	0.794	0.730	0.730	0.794
HBME-1	0.794	0.757	0.750	0.800
CK19	0.765	0.730	0.722	0.771

Gal-3-Galectin-3; Ret-Ret oncoprotein; CK 19-Cytokeration 19; PPV-Positive predictive value; NPV-Negative predictive value

A further review of the results in Table 2 reveals that immunoexpression of the four markers had a somewhat distinct pattern among the three groups of nonneoplastic (HN), benign neoplastic (FA/HA), and malignant. While only about a quarter of the nonneoplastic lesions showed immunoexpression for each of the four markers, about half of the benign neoplastic lesions and close to 90% of the malignant tumors were immunoreactive for each of the markers.

Table 6 depicts the results of immunoexpression of various combinations of the four tested markers. The results show that the panel of galectin-3 + HBME-1 has the highest sensitivity and specificity of immunoexpression in all benign lesions versus malignant tumors, 90.7% and 75%, respectively. However, this is lower than that of galectin-3 values (92.6% and 77.3%, respectively). Additionally, these results were not statistically significant with *P* value >0.05 for the galectin-3 + HBME-1 in differentiating all benign lesions from malignant tumors. Also, the PPV and NPV for these combination panels are lower than those of the single markers including galectin-3.

**Table 6:** Comparison of sensitivity, specificity, PPV, and NPV of combined markers expression in all benign versus malignant thyroid lesions

*Markers*		*Negative*	*Positive*	*Total*	*Combo values*	
Gal-3 + HBME-1	Benign	66	22	88	Sensitivity	0.907
	Malignant	5	49	54	Specificity	0.750
					PPV	0.690
Total		71	71	142	NPV	0.930
Gal-3 + CK19	Benign	61	20	81	Sensitivity	0.836
	Malignant	10	51	61	Specificity	0.753
					PPV	0.718
Total		71	71	142	NPV	0.859
HBME-1 +CK19	Benign	59	22	81	Sensitivity	0.820
	Malignant	11	50	61	Specificity	0.728
					PPV	0.694
Total		70	72	142	NPV	0.843
Gal-3 + HBME +	Benign	93	32	125	Sensitivity	0.852
CK19	Malignant	13	75	88	Specificity	0.744
					PPV	0.701
Total		106	107	213	NPV	0.877

Gal-3-Galectin-3; Ret-Ret oncoprotein; CK 19-Cytokeratin 19; PPV-Positive predictive value; NPV-Negative predictive value

## DISCUSSION

Thyroid nodules are common lesions in clinical practice, but only a minority is malignant or suspicious tumors that require surgical resection. The decision for the surgical excision of thyroid nodules is based on clinical information, thyroid ultrasound and scintigraphy, and FNA diagnosis. Among these, FNA is considered the most effective method of identifying patients requiring surgical intervention.[1,2,6–9,23,31] In fact, the present consensus is that thyroid FNA biopsy is the procedure of choice for the evaluation of these nodules with a proven track record of good diagnostic value and accuracy. Furthermore, recent advances including a widespread use of the liquid-based monolayer cytopreparation of thyroid FNA specimens have increased the diagnostic yield and accuracy and decreased the unsatisfactory rate. Most recently, the recommendation for the performance of thyroid FNA under ultrasound imaging guidance and growing utilization of this technique have also improved the yield and accuracy of the procedure.[32] Nevertheless, there are some inherent limitations in thyroid FNA, and cytopathologists sometimes still encounter difficulties in rendering accurate diagnosis. In fact, one frequent dilemma that faces cytopathologists is the inability to differentiate benign from malignant follicular tumors, because the distinction is based strictly on the presence or absence of vascular and/or capsular invasion.[33] Another problem is distinguishing between FVPC and follicular neoplasm on thyroid FNA samples.[3–5,34] Up to now, no effective method has been established to accurately distinguish these thyroid lesions in the presurgical phase.

For the aforementioned reasons, there have been increasing attempts during the last decade to identify molecular and IHC markers that can help in making a more accurate diagnosis of thyroid nodules. Studies have been conducted mostly on surgically resected thyroid specimens to evaluate the utility of various IHC markers in separating benign lesions that can treated medically, from malignant tumors that require surgical intervention. However, similar studies were also conducted on FNA cytologic specimens using cell block preparations.[22,27] Other investigations were performed on cytology smears, prepared mostly with the liquid-based monolayer technique (ThinPrep slides), although some have been conducted on destained Diff-Quik or Papanicolaou-prepared direct smears. Nasser *et al.* used various cytology preparations to evaluate markers expression in thyroid FNAs and found that smears made using methanol-fixed thin-layered slides had the strongest reaction and best results.[18] In general, the studies have shown similar results of markers' expression between surgical specimens and FNA cell block sections. As a result of investigating a large number of IHC markers, a few have emerged as potentially useful for differentiating benign from malignant thyroid nodules, including galectin-3, CK19, HBME-1, and Ret oncoprotein. Based on this information, we chose to test these most promising markers on a histologically proven thyroid lesions initially diagnosed on FNABs.

Defining the cut-off limit for considering a case as positive has varied among investigators. While some considered any degree of staining as positive, others have demanded 5% or 10% or more reactivity to define a case as positive. We chose a strict limit of > 10% reactivity to consider a case as positive.

Our study showed that it is feasible to evaluate immunoexpression of several IHC markers on cell block sections of thyroid FNA, as was also observed by other investigators.[14,27] Our results concurred in essence with the findings of previous authors that these markers, especially galectin-3 and HBME-1, are helpful as an adjunct ancillary method for establishing more definitive diagnosis when dealing with follicular-derived thyroid lesions. The immunoexpression, however, must be used in addition to, and not in lieu of, established cytologic criteria. We think that these results could be very helpful in the setting of thyroid nodules showing cytologic features of follicular neoplasm on FNA. Positive immunostaining of these markers especially galectin-3 and HBME-1 would be further in support of a follicular carcinoma rather than follicular adenoma. However, because of the apparent overlap in markers expression, one cannot, at least for now, be absolutely certain that positive immunoexpression by itself is sufficient to confirm a follicular neoplasm lesion on FNA as FC. Additional studies evaluating IHC expression of a large number of follicular neoplasms on FNAs with available subsequent surgical confirmation are needed to further validate our findings.

Unfortunately, the number of cases of the specific malignancies (FC, PTC, FVPC) is too small to make a meaningful statistical comparison. Specifically, we cannot make a strong conclusion whether the immunoexpression of these markers is helpful to discriminate between FC (six cases) and FVPC (two cases). Again, larger studies dedicated to evaluate the role of these or other markers for the distinction between FC and FVPC can be particularly useful.

Our data show that there is a distinct and clear difference of immunoexpression for all four markers with a progressive increase in the staining rate from nonneoplastic to benign to malignant tumors [Table 2]. This is essentially in agreement with previous studies, although our data show higher expression in nonneoplastic and benign neoplastic lesions than that observed in other studies. We speculate that this may be due in part to the use of the avidin-biotin complex detection system which could react with endogenous biotin-like substances. Therefore, the use of biotin-free detection system could help to avoid interfering technical factors (endogenous biotin-like activity). Other technical and procedural parameters such as fixation, antigen retrieval, or commercial antibodies' clones used may play a role.

As shown in Table 2, nonneoplastic lesions demonstrated very low immunoexpression for all four markers with the lowest level being noted in galectin-3 (8/37, 2l.6%). In general, about one-quarter of these lesions was positive for the tested IHC markers. Macrophages stained strongly with galectin-3, and sometimes obscured the follicular cells in cases of HN with prominent cystic component. Therefore, positivity of follicular cells in these cases may be false due to carry-over of the associated abundant macrophages. Follicular adenomas showed intermediate expression of all markers between HN and malignant tumors ranging from 28.5% for galectin-3 to 57.1% for Ret. Both HBME-1 and CK19 had 42.9% reactivity. Table 3 depicts the values of sensitivity, specificity, PPV, and NPV for distinguishing all benign lesions from malignant tumors for the tested markers.

Galectin-3 showed the highest immunoexpression in malignant tumors (25/27, 92.6%) and lowest reaction in benign lesions (10/44, 22.7%). Furthermore, this marker had the best sensitivity and specificity in differentiating benign from malignant nodules with values of 92.6% and 77.3%, respectively [Table 2]. Previous studies revealed similar data and recommended the use of this marker as a useful test to identify thyroid malignancies including FC and PTC.[16,19,20] Orlandi *et al.* and Saggiorato *et al.* recommended the application of galectin-3 as a useful IHC test for presurgical diagnosis of thyroid nodules that require surgical removal.[23,25]

HBME-1 also had a high immunoexpression level in malignant tumors (24/27, 88.8%) compared to benign lesions (12/44, 27.3%). This is in agreement with previous studies showing a high rate of immunoexpression of HBME-1 in malignant thyroid tumors.[21,22,24] A study by Sack *et al.* concluded that HBME-1 positivity in FNA of thyroid nodules supports the diagnosis of carcinoma, and Nga *et al.* found that a panel of HBME-1 and CK19 is highly discriminatory in the diagnostic workup for PTC.

CK19 and Ret showed closely similar values in benign and malignant lesions and were clearly lower than those of galectin-3. Results of previous studies in this regard found strong reactivity of this marker in papillary thyroid carcinomas but not follicular adenoma or carcinoma.[13,18] It is known that thyroid tumor progression is modulated by multiphase oncogene activation. Thyroid malignancies not expressing markers can be assumed to be due to technical errors in fixation procedures, or sampling error during FNA procedure.

In practical terms, we have observed that marker combinations did not improve the detection of malignancy beyond that obtained by galectin-3 alone [Table 6]. This is in contrast with results of other studies that showed higher sensitivity and specificity when using a panel of two or more markers.[11,26] However, in our experience, the use of a panel of two markers is recommended to overcome technical errors or staining problems and to eliminate the delay in diagnosis.

## CONCLUSION

We conclude that IHC staining is of value as an ancillary test to enhance the diagnostic accuracy of thyroid nodules on FNA biopsy. Our results indicate that galectin-3 is the most sensitive and specific single marker in differentiating benign from malignant nodules, seconded by the HBME-1 marker. We recommend the use of a small panel of galectin-3 and HBME-1 as an adjunct to standard cytomorphology criteria to enhance the diagnostic accuracy of thyroid nodules with follicular-patterned cytologic features.

## COMPETING INTEREST STATEMENT BY All AUTHORS

No competing interest to declare by any of the authors.

## STATEMENT BY ALL AUTHORS

All authors have participated in various parts of this study including the design, preparation, review of materials, writing, and review of the manuscript.

## ETHICS STATEMENT BY ALL AUTHORS

This study was conducted with approval from the Institutional Review Board (IRB) of all the institutions associated with the study.
